# Percutaneous calcium phosphate cement injection for unicameral bone cysts: mid- to long-term clinical and radiographic outcomes

**DOI:** 10.3389/fsurg.2026.1900796

**Published:** 2026-08-28

**Authors:** Mengzhang Xie, Zhengxiao Lai, Yi Luo, Li Min, Chongqi Tu, Yong Zhou

**Affiliations:** 1 Department of Orthopedics, Orthopaedic Research Institute, West China Hospital, Sichuan University, Chengdu, China; 2 Model Worker and Craftsman Talent Innovation Workshop of Sichuan Province, West China Hospital, Sichuan University Chengdu, China; 3 Anesthesiology and Operation Center, West China Tianfu Hospital, Sichuan University, Chengdu, Sichuan, China

**Keywords:** calcium phosphate cement, minimally invasive treatment, percutaneous injection, recurrence, unicameral bone cyst

## Abstract

**Objective:**

Unicameral bone cysts (UBCs) are fluid-filled, osteolytic lesions commonly affecting children and adolescents. Minimally invasive treatments such as percutaneous injection have become increasingly popular. Calcium phosphate cement (CPC), a biocompatible artificial bone substitute with mechanical strength and osteoconductivity, and may be suitable for such applications. This study aimed to evaluate the mid- to long-term clinical and radiographic outcomes of percutaneous calcium phosphate cement injection for UBCs, with particular emphasis on late recurrence, repeat injection, cortical remodeling, and cavity filling.

**Methods:**

A retrospective review was conducted on 31 patients with UBC treated by CPC injection between January 2013 and January 2025, with at least 1 year of follow-up. Postoperative outcomes were assessed radiographically, functionally, and for complications. We recorded the patients’ age, sex, preoperative and postoperative visual analog scale (VAS) scores, Musculoskeletal Tumor Society (MSTS) scores, modified Neer classification and Capanna classification, cortical thickness ratio, cavity filling, recurrence, repeat intervention, and complications.

**Results:**

The mean follow-up duration was 61.1 months (range, 13–149 months). Among them, 14 patients experienced pathological fractures. Postoperatively, pain and functional outcomes improved significantly, with the median VAS score decreasing to 0 and the median MSTS score improving to 30 at final follow-up (*P* < 0.001). Recurrence after the first injection occurred in four patients (12.9%). Among them, three patients underwent repeat percutaneous CPC injection, and one patient was managed with observation because of adequate cortical thickness and clinical stability. At final follow-up after all treatments, all patients achieved modified Neer grade I or II outcomes, and most patients achieved Capanna grade I or II healing. One patient experienced delayed wound healing, and no severe complications were observed.

**Conclusion:**

Percutaneous CPC injection was associated with pain relief, functional improvement, and radiographic stabilization in patients with UBCs. Repeat CPC injection appeared to provide stable outcomes in recurrent cases, whereas observation was considered reasonable for one clinically stable lesion with adequate cortical thickness. The observed cortical remodeling and cavity filling suggest that CPC injection may represent a potential minimally invasive strategy for reconstruction of UBC-related bone defects.

## Introduction

Unicameral bone cysts (UBCs), also known as simple bone cysts, are benign, solitary, fluid-filled osteolytic lesions. When partial internal septations are present, the lesion may be described as a partially septated unicameral bone cyst. UBCs are relatively common, accounting for approximately 3% of all bone tumors. They predominantly occur in growing children and adolescents, with a peak incidence between 3 and 14 years of age and a male-to-female ratio of 2:1 (1–3). Approximately 75% of UBCs are found in the proximal regions of long bones, particularly the humerus and femur. Less common sites include the calcaneus, tibia, and fibula. UBCs have also been reported in rarer locations, such as the pelvis, cranial vault, mandible, and lunate bone (4–8). Approximately 80% of UBCs are asymptomatic and are often discovered incidentally during physical examinations following intense physical activity. However, when pathological fractures are present, symptoms such as pain and restricted mobility may occur.

The management of UBCs, which are benign tumor-like lesions, should be tailored to factors such as cyst size, location, symptoms, and the risk of pathological fractures. The primary treatment goals are to strengthen the affected bone, prevent fractures, alleviate symptoms, minimize recurrence, and reduce the risk of secondary fractures. Currently, there is no universally accepted treatment strategy for UBCs. Treatment approaches generally fall into three categories: conservative management, open surgery, and minimally invasive procedures. For patients with asymptomatic UBCs that are incidentally discovered and carry a low risk of pathological fractures, conservative management through regular monitoring is recommended. However, surgical intervention is indicated for patients with existing pathological fractures, a high risk of fracture, or symptoms that significantly impact daily life, particularly when conservative measures prove ineffective. The main surgical objective in treating UBCs is to remove the lesion, reduce the likelihood of recurrence, and reconstruct the resulting bone defect. Open surgical techniques include lesion curettage combined with bone grafting, internal fixation via plates or intramedullary nails, and fenestration decompression (9, 10). Minimally invasive surgical techniques for treating UBCs include elastic stable intramedullary nailing (ESIN), percutaneous aspiration and injection therapy, and percutaneous decompression (11–13). Among these methods, injection therapy is the simplest and quickest procedure. According to a systematic review and meta-analysis of 4,973 UBC cases, 35.9% of patients underwent injection therapy, making it the second most common treatment option after curettage (8). Injection therapy promotes cyst healing by delivering therapeutic substances directly into the cyst cavity. Substances commonly used include methylprednisolone acetate (MPA), demineralized bone matrix (DBM), *α*-tricalcium phosphate bone substitute material (*α*-BSM), and bone morphogenetic protein (BMP) (14, 15).

Artificial bone is a biomaterial used to replace, repair, or promote the regeneration of bone tissue and has been increasingly used for the reconstruction of bone defects. Injectable bone cement, a form of artificial bone, can be injected and solidified within the defect, providing mechanical support while promoting new bone formation. A key requirement for this material is that it must be set at physiological temperatures without releasing excessive heat (16). Calcium phosphate bone substitutes are primarily composed of calcium ions and phosphate anions, which are the main inorganic components of natural bone, thus offering excellent biocompatibility (17). CPC is a widely used application of calcium phosphate artificial bone and is known for its good biocompatibility, osteoconductivity, and self-curing properties, setting without generating significant heat (18). Compared with calcium sulfate-based cements and polymethyl methacrylate (PMMA), CPC has superior new bone formation potential (19). Reports on artificial bone treatment for UBCs often involve calcium sulfate-based bioceramics. These materials degrade quickly but have lower mechanical strength than CPC bone cement does. In contrast, self-curing calcium phosphate bone cements degrade more slowly, offering greater mechanical strength, making them suitable for long-term mechanical support in bone defect repairs. Minimally invasive surgery is generally more acceptable to patients; thus, CPC bone cement is emerging as an attractive treatment option.

Although several injectable bone substitutes have been used for UBCs, most available studies have focused on short- to mid-term healing or early recurrence. Evidence regarding late recurrence, the durability of radiographic remodeling, and the outcome of repeat CPC injection remains limited. Therefore, this study retrospectively evaluated the mid- to long- term clinical and radiographic outcomes of percutaneous CPC injection for UBCs, focusing on recurrence, repeat injection, cortical remodeling, cavity filling, and functional recovery.

## Patients and methods

### Patient selection and ethical clearance

This study was conducted in accordance with the principles outlined in the Declaration of Helsinki and approved by the Institutional Review Board of Sichuan University West China Hospital. Written informed consent was obtained from all patients prior to their participation in the study.

Between January 2013 and January 2025, we retrospectively reviewed 31 patients with UBCs who underwent percutaneous CPC injection at our institution. All patients met the following criteria: 1) diagnosis of UBC confirmed by histopathological examination; 2) presence of surgical indications, including pain, progressive lesion enlargement, cortical thinning, pathological fracture, or high risk of fracture; 3) treatment with percutaneous CPC injection; and 4) availability of at least 12 months of clinical and radiographic follow-up data. Patients with severe surgical contraindications, lesions other than UBCs, or incomplete clinical and imaging data were excluded.

### Preoperative preparation

Preoperative examinations, including necessary x-rays, computed tomography (CT) scans, thin-slice CT of the lungs and magnetic resonance imaging (MRI) were conducted to exclude surgical contraindications. Additionally, auxiliary examinations, combined with the patient's medical history, were used to establish a clear diagnosis. For benign tumor lesions, whether to perform a preoperative biopsy or proceed directly with surgery was discussed with the patient. After patients’ consent was obtained, the appropriate treatment plan was implemented. On digital radiographs, the maximum longitudinal diameter (L) and the maximum transverse diameter perpendicular to L (H) were measured on the anteroposterior view, whereas the maximum anteroposterior diameter (W) was measured on the lateral view. The cyst was approximated as an ellipsoid, and the estimated radiographic cyst volume was calculated using the formula (π × L × H × W)/6. Cysts were classified as active when the shortest distance between the cyst margin and the adjacent physis was less than 10 mm, and as latent when this distance was 10 mm or greater (20). To assess preoperative function, the Musculoskeletal Tumor Society (MSTS) score and visual analog scale (VAS) score were used. The MSTS score consists of upper and lower limb assessments and considers multiple dimensions, such as pain, function, and emotional acceptance. The evaluation of functional outcomes following percutaneous CPC injection therapy is divided into 6 dimensions, with a maximum score of 5 points per dimension, for a total of 30 points. VAS is a commonly used tool for assessing pain intensity and is scored from 0 to 10. It is particularly useful for younger patients, as it allows them to accurately mark their pain intensity on the scale, transforming subjective pain experiences into quantitative data.

### Surgical technique

**Figure 1:**
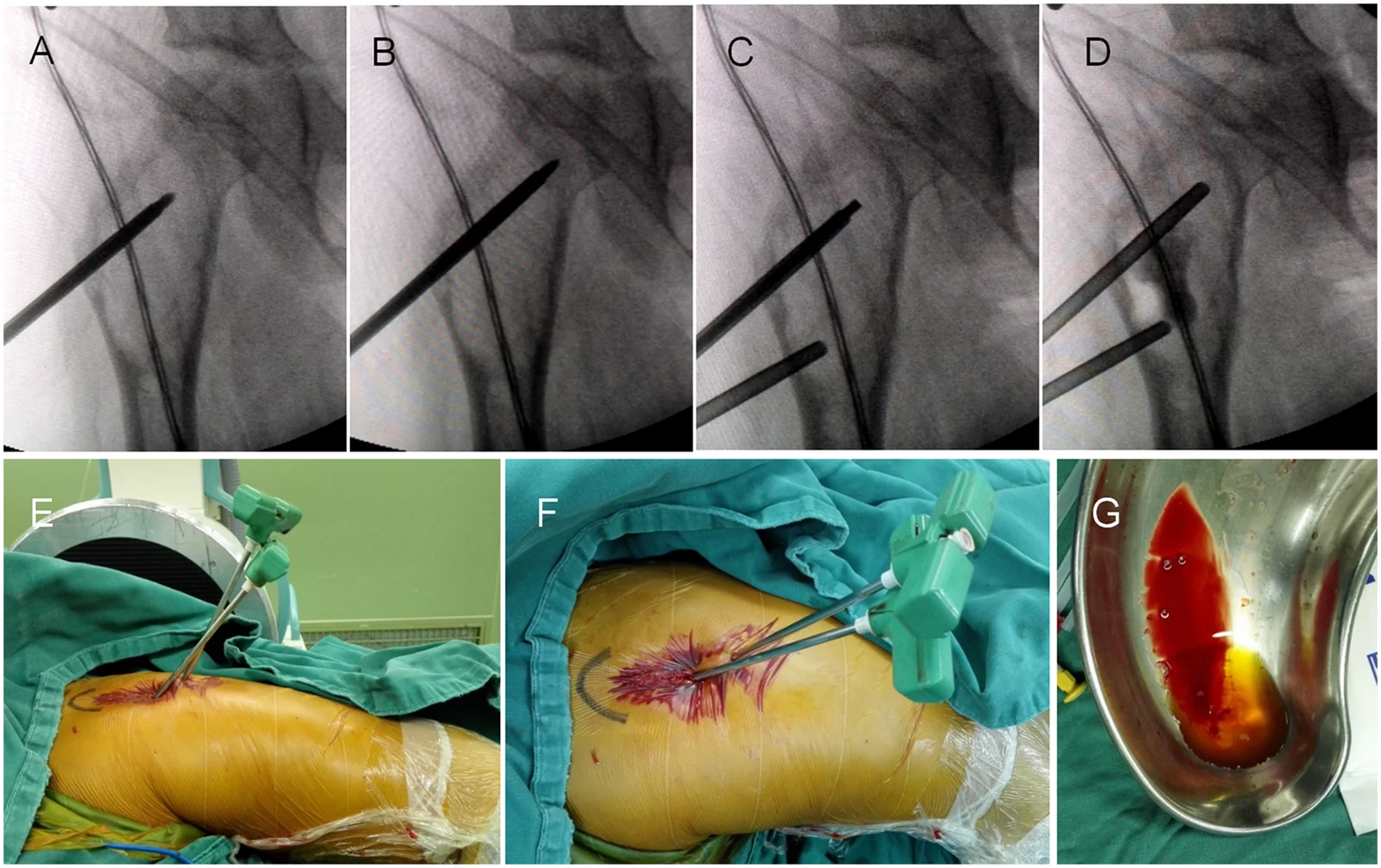
Intraoperative photon. **(A–D)** intraoperative puncture and injection under the guidance of C-arm; **(E,F)** intraoperative general appearance photos; **(G)** Bone cyst lesion Hemorrhagic fluid extracted by puncture.

All surgeries were performed under general anesthesia by two orthopedic surgeons specializing in musculoskeletal oncology. Following anesthesia, the patient was positioned appropriately on the basis of the lesion location, and routine disinfection and draping were carried out. Under C-arm fluoroscopic guidance, two longitudinal incisions (approximately 0.5–1 cm each) were made above and below the lesion site, cutting through the skin and subcutaneous layers, with careful dissection of the muscle and other soft tissues down to the bone surface. Two bone biopsy needles were then inserted into the lesion cavity under C-arm guidance. Hemorrhagic fluid was aspirated, and one biopsy needle served as the inlet while the other served as the outlet. The cavity was thoroughly irrigated with copious amounts of normal saline. Following irrigation, artificial bone was prepared and injected into the lesion cavity until satisfactory filling was achieved (Figure 1). The surgical area was extensively irrigated again, the instruments were counted, and the puncture channels were covered with a well-formed local fascial flap. Finally, the skin was sutured, and the wound was disinfected and dressed.

### Postoperative management and evaluation

After surgery, patients are advised to have monthly follow-ups for the first three months, then biannual follow-ups until one-year postoperation, and annual assessments thereafter. The main postoperative evaluations include imaging, functional, and complication assessments.

Imaging evaluation: Radiographic measurements were performed by two experienced radiologists. The measurement results and radiographic classifications were then reviewed by two orthopedic surgeons specializing in musculoskeletal oncology to confirm their clinical and anatomical accuracy. Any discrepancies were resolved through discussion until consensus was reached. Each follow-up involves x-ray evaluations of the lesion via the modified Neer classification (21) (Table 1) and Capanna classification (22) (Table 2). Bone cortex thickness deficits can increase the risk of pathological fractures. However, as Peter Frederik Horstmann (23) suggested, minor reductions in cortical thickness might not impact young, healthy individuals, so cortical thickness alone is not sufficient as an indicator of bone strength. Instead, the cortical thickness ratio (the volume of cortical bone in the lesion compared with the surrounding normal cortex) and cavity filling (the proportion of normal bone tissue present at the original site of the lesion) should be measured. On the basis of their measurement methodology, we assessed cortical thickness and cavity filling via x-ray images. In this study, radiographic recurrence was determined according to the modified Neer classification together with dynamic changes on serial follow-up radiographs. Therefore, recurrence was not determined based on a single radiographic assessment alone but was assessed by considering whether a cystic lesion reappeared in a previously healed area or whether a residual radiolucent area increased in size during follow-up.

**Table 1:** Modified Neer classification.

Description	Classification	Score
Cyst filled by formation of new bone with or without small static, radiolucent area(s) less than 1 cm in size	Healed	I
Static, radiolucent area(s) less than 50% of the diameter of bone with enough cortical thickness to prevent fracture	Healing with defect	II
Radiolucent areas greater than 50% of diameter of the bone and with a thin cortical rim. No increase in cyst size. Continued restriction of activity or repeated treatment is required	Persistent cyst	III
Cyst reappeared in a previously obliterated area or a residual radiolucent area has increased in size	Recurrent cyst	IV

**Table 2:** Capanna classification.

Description	Classification	Score
The cyst completely filled in with bone, and the cortical margin thickened	Healed	I
The cyst was consolidated with bone and the cortical margin thickened, but there were still small residual areas of osteolysis within the cyst	Healed with residual	II
Radiolucent areas greater than 50% of diameter of the bone and with a thin cortical rim. No increase in cyst size. Continued restriction of activity or repeated treatment is required	Recurrence	III
The cyst showed no evidence of response to treatment.	No response	IV

For statistical analysis, both cavity filling and the cortical thickness ratio were categorized into four groups: >90%, 50%–90%, 10%–50%, and <10%. The modified Neer classification categorizes results into “Healed,” “Healing with defect,” “Persistent cyst,” and “Recurrent cyst” (Table 1), whereas the Capanna classification assesses responses as “Healed,” “Healed with residual,” “Recurrence,” and “No response,” with Roman numerals I–IV representing these four categories. Both classifications indicate treatment failure at grade III or IV, with Capanna emphasizing the response of the cyst to treatment and modified Neer focusing on the presence and size changes of the cyst.

Functional evaluation: MSTS and visual analog scale (VAS) scores were recorded at each follow-up to assess postoperative function.

Complication assessment: We monitored signs of wound inflammation, infection, recurrence, pathological fractures, and material rejection to evaluate surgical complications.

Follow-up was performed according to the recommended schedule whenever possible; however, actual follow-up intervals varied in this retrospective cohort. Intermediate radiographic data that were unavailable at specific scheduled time points were not imputed, and radiographic outcomes were analyzed based on the available follow-up records. All included patients had at least 12 months of radiographic follow-up, and final follow-up data were available for all patients included in the analysis.

### Statistical analysis

Continuous variables, including age, follow-up duration, and cyst size, were summarized as medians with interquartile ranges or ranges, as appropriate. Categorical variables, including sex, lesion location, cyst activity, pathological fracture, recurrence, modified Neer classification, Capanna classification, cavity filling, and cortical thickness ratio, were presented as frequencies and percentages. Preoperative and final follow-up VAS and MSTS scores were compared using the Wilcoxon signed-rank test. An exploratory subgroup analysis was performed to compare patients with and without preoperative pathological fracture. Fisher's exact test was used for categorical variables, and the Mann–Whitney U test was used for continuous or ordinal variables between groups. A *post-hoc* sensitivity analysis was performed to estimate the minimum detectable effect size. A two-sided *P* value <0.05 was considered statistically significant. All statistical analyses were performed using SPSS version 27.0 (IBM, New York, USA).

## Results

### Patient characteristics

A total of 31 patients with UBCs who underwent percutaneous CPC injections were included in this study. There were 24 males and 7 females. The median age at surgery was 10.0 years (interquartile range [IQR], 7.5–12.5 years; range, 3.0–30.3 years). The mean follow-up duration was 61.1 months (range, 13–149 months). Twenty-five patients (80.6%) had more than 3 years of follow-up, 11 patients (35.5%) had more than 5 years of follow-up, and 4 patients (12.9%) had more than 10 years of follow-up (Table 3).

**Table 3:** Baseline characteristics of patients.

Case	Age (years)	Sex	Location	Follow-up (months)	Previous pathological fracture	Previous treatment	Preoperative VAS score	Preoperative MSTS score	Cyst size (cm^3^)	Cortex thickness ratio
1	11.6	M	Proximal femur	139	No	No	3	27	70.98	＜10%
2	13.9	F	Humeral shaft	131	Yes	conservative	4	20	16.28	＜10%
3	19.5	M	Proximal femur	37	Yes	conservative	3	26	41.43	＜10%
4	6.5	M	Proximal femur	115	No	No	2	27	14.5	＜10%
5	10.6	M	Proximal femur	59	No	surgical	4	24	22.95	＜10%
6	6.1	M	Proximal humerus	36	Yes	conservative	4	26	11.05	10–50%
7	25.6	F	Proximal humerus	137	No	No	2	28	20.55	10–50%
8	9	M	Proximal humerus	84	No	No	3	27	12.4	＜10%
9	30.3	M	Calcaneus	38	No	No	4	26	67.26	10–50%
10	12	M	Proximal humerus	36	Yes	conservative	4	26	16.73	＜10%
11	6	M	Proximal humerus	69	Yes	surgical	6	13	19.8	10–50%
12	8	M	Proximal humerus	87	No	No	4	22	5.8	50–90%
13	7	M	Proximal humerus	37	Yes	conservative	4	23	9.3	＜10%
14	11	M	Proximal humerus	76	Yes	conservative	4	22	9.6	50–90%
15	23	M	Calcaneus	51	No	No	4	24	115.55	10–50%
16	9.4	F	Distal fibula	38	No	No	5	22	2.98	＜10%
17	10.4	M	Proximal humerus	149	No	No	4	23	76.07	＜10%
18	9.7	M	Proximal femur	36	Yes	conservative	4	22	14.48	＜10%
19	6	F	Proximal humerus	55	No	No	3	26	18.8	＜10%
20	15	F	Proximal humerus	49	No	No	2	27	9.62	＜10%
21	3	M	Distal fibula	43	No	No	4	26	5.62	＜10%
22	13	M	Pelvis	40	No	No	3	28	12.5	＜10%
23	12	F	Proximal humerus	37	No	No	2	28	8.9	＜10%
24	15	M	Proximal humerus	47	Yes	surgical	1	28	16.21	＜10%
25	12	F	Proximal femur	67	Yes	surgical	3	27	24.63	＜10%
26	6	M	Proximal femur	22	No	surgical	2	28	20.2	10–50%
27	10	M	Proximal humerus	13	Yes	conservative	2	29	10.5	10–50%
28	7	M	Proximal humerus	40	Yes	conservative	4	27	9.84	＜10%
29	8	M	Proximal humerus	48	Yes	conservative	3	27	10.13	＜10%
30	9	M	Distal fibula	66	Yes	conservative	3	28	4.8	10–50%
31	9	M	Proximal femur	13	No	No	4	26	42.34	＜10%

The lesions were in the proximal humerus in 16 patients, proximal femur in 8 patients, distal fibula in 3 patients, calcaneus in 2 patients, humeral shaft in 1 patient, and pelvis in 1 patient. Fourteen patients had a previous pathological fracture before CPC injection. Fifteen patients had no previous treatment, while the remaining patients had received splint immobilization, cast immobilization, sling immobilization, internal fixation, or curettage and bone grafting before CPC injection. The median cyst size was 14.50 cm^3^(IQR, 9.73–21.75; range, 2.98–115.55). Preoperatively, the cortical thickness ratio was <10% in 21 patients, 10%–50% in 8 patients, and 50%–90% in 2 patients.

### Clinical outcomes

Pain and functional outcomes improved after treatment. The median VAS score decreased from 4 preoperatively (IQR, 3–4; range, 1–6) to 0 at final follow-up (IQR, 0–0; range, 0–2), and the difference was statistically significant (*P* < 0.001). The median MSTS score improved from 26 preoperatively (IQR, 23.5–27; range, 13–29) to 30 at final follow-up (IQR, 29–30; range, 28–30), and the improvement was also statistically significant (*P* < 0.001). At the final follow-up, most patients had no residual pain and had resumed satisfactory daily activity.

### Final radiographic outcomes

Final clinical outcomes and radiographic structural parameters after all treatments are summarized in Table 4. At final radiographic assessment, 16 patients were classified as modified Neer grade I and 15 patients as grade II. No patient was classified as modified Neer grade III or IV at final follow-up. According to the Capanna classification, 15 patients were grade I, 15 patients were grade II, and 1 patient was grade III.

**Table 4:** Final clinical outcomes and radiographic structural parameters after all treatments.

Case	Recurrence after first injection	No. of injections	Complications	Final VAS score	Final MSTS score	Final cortical thickness ratio	Final cavity filling
1	Yes	2	No	2	29	50%–90%	50%–90%
2	No	1	No	0	29	50%–90%	>90%
3	No	1	No	0	29	50%–90%	50%–90%
4	No	1	Delayed wound healing	0	30	50%–90%	>90%
5	No	1	No	1	29	10%–50%	50%–90%
6	No	1	No	0	29	>90%	>90%
7	No	1	No	0	30	50%–90%	>90%
8	No	1	No	0	30	>90%	>90%
9	No	1	No	1	29	50%–90%	50%–90%
10	No	1	No	0	29	>90%	50%–90%
11	No	1	No	0	29	>90%	>90%
12	No	1	No	0	30	>90%	>90%
13	No	1	No	0	29	>90%	>90%
14	No	1	No	0	29	50%–90%	50%–90%
15	No	1	No	0	30	>90%	>90%
16	No	1	No	0	29	>90%	50%–90%
17	Yes	2	No	0	30	50%–90%	>90%
18	No	1	No	0	29	>90%	>90%
19	Yes	2	No	2	28	>90%	>90%
20	No	1	No	0	30	>90%	>90%
21	No	1	No	0	29	>90%	>90%
22	No	1	No	0	30	>90%	>90%
23	No	1	No	0	30	>90%	>90%
24	No	1	No	0	30	>90%	>90%
25	No	1	No	0	30	>90%	>90%
26	No	1	No	0	30	>90%	50%–90%
27	No	1	No	1	30	>90%	50%–90%
28	No	1	No	0	30	>90%	>90%
29	Yes	1	No	2	29	50%–90%	50%–90%
30	No	1	No	0	30	>90%	>90%
31	No	1	No	0	30	>90%	50%–90%

**Figure 2:**
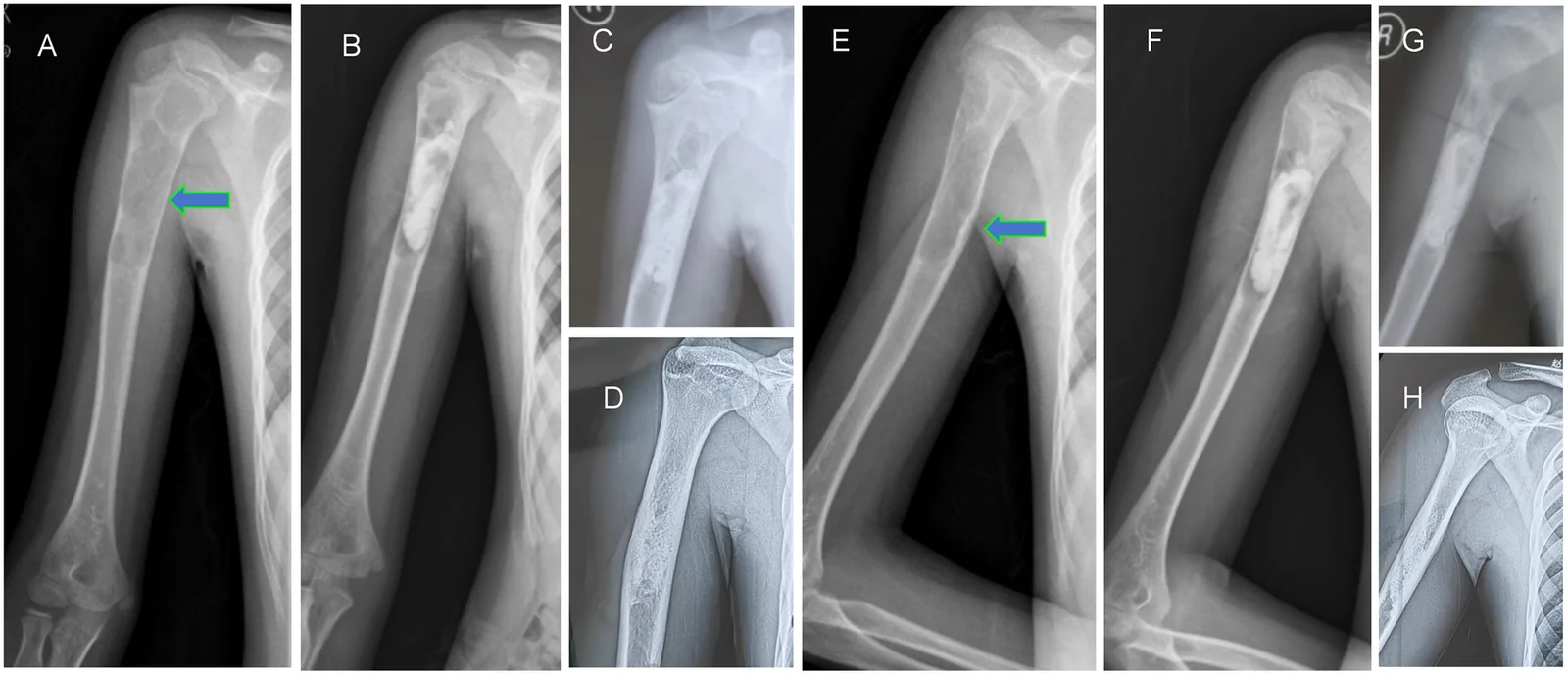
Representative radiographic changes in patient 8 after a single percutaneous CPC injection. **(A)** Preoperative anteroposterior (AP) radiograph showing the lesion, indicated by the arrow. **(B–D)** Postoperative AP radiographs obtained at 3 months, 1 year, and 7 years after injection, respectively. **(E)** Preoperative lateral radiograph showing the lesion, indicated by the arrow. **(F–H)** Postoperative lateral radiographs obtained at 3 months, 1 year, and 7 years after injection, respectively. Progressive cavity consolidation and cortical remodeling were observed during follow-up.

The final cortical thickness ratio was >90% in 21 patients, 50%–90% in 9 patients, and 10%–50% in 1 patient. Final cavity filling was >90% in 20 patients and 50%–90% in 11 patients. No patient had final cavity filling <50%. Serial radiographic outcomes after the first percutaneous CPC injection are shown in Table 5. Representative case after a single CPC injection demonstrated progressive cortical thickening and cavity consolidation during long-term follow-up (Figure 2). At final radiographic assessment, most patients maintained satisfactory cortical thickness and cavity filling.

**Table 5:** Serial radiographic outcomes after the first percutaneous calcium phosphate cement injection.

Case	3 months	1 year	2 years	3 years	5 years	7 years	10 years	Final radiographic assessment after first injection
1*	Neer II/Capanna II	Recurrent: Neer IV/Capanna IV	—	—	—	—	—	Recurrent at 1 year; see Table 6
2	Neer II/Capanna II	Neer I/Capanna II	Neer I/Capanna II	Neer II/ Capanna II	Neer II/ Capanna II	Neer II/ Capanna II	Neer II/ Capanna II	Neer II/Capanna II
3	Neer I/Capanna I	Neer I/Capanna I	Neer I/Capanna I	Neer I/Capanna I	NA	NA	NA	Neer I/Capanna I
4	Neer I/Capanna I	Neer I/Capanna I	Neer I/Capanna I	Neer I/Capanna I	Neer I/Capanna I	Neer I/Capanna II	NA	Neer I/Capanna II
5	Neer II/Capanna II	Neer II/Capanna II	Neer II/Capanna II	Neer II/Capanna II	Neer II/Capanna II	NA	NA	Neer II/Capanna II
6	Neer II/Capanna II	Neer II/Capanna II	Neer II/Capanna II	Neer II/Capanna II	NA	NA	NA	Neer II/Capanna II
7	Neer I/Capanna I	Neer I/Capanna I	Neer I/Capanna I	Neer I/Capanna II	Neer I/Capanna II	Neer I/Capanna II	Neer II/Capanna II	Neer I/Capanna II
8	Neer I/Capanna I	Neer II/Capanna II	Neer II/Capanna II	Neer II/Capanna II	Neer II/Capanna II	Neer II/Capanna II	NA	Neer II/Capanna II
9	Neer I/Capanna I	Neer I/Capanna I	Neer I/Capanna I	Neer I/Capanna I	NA	NA	NA	Neer I/Capanna I
10	Neer II/Capanna II	Neer II/Capanna II	Neer II/Capanna II	Neer II/Capanna II	NA	NA	NA	Neer II/Capanna II
11	Neer I/Capanna I	Neer I/Capanna I	Neer I/Capanna I	Neer I/Capanna II	Neer II/Capanna II	NA	NA	Neer II/Capanna II
12	Neer I/Capanna I	Neer I/Capanna I	Neer I/Capanna I	Neer I/Capanna I	Neer I/Capanna I	Neer I/Capanna II	NA	Neer I/Capanna II
13	Neer I/Capanna I	Neer I/Capanna I	Neer I/Capanna I	Neer I/Capanna I	NA	NA	NA	Neer I/Capanna I
14	Neer I/Capanna II	Neer I/Capanna II	Neer II/Capanna II	Neer II/Capanna II	Neer II/Capanna II	NA	NA	Neer II/Capanna II
15	Neer I/Capanna I	Neer I/Capanna I	Neer I/Capanna I	Neer II/Capanna II	NA	NA	NA	Neer II/Capanna II
16	Neer I/Capanna II	Neer I/Capanna II	Neer I/Capanna II	Neer II/Capanna II	NA	NA	NA	Neer II/Capanna II
17*	Neer I/Capanna I	Neer I/Capanna I	Neer II/Capanna III	Recurrent: Neer IV/Capanna IV	—	—	—	Recurrent at 3 years; see Table 6
18	Neer I/Capanna I	Neer I/Capanna I	Neer II/Capanna II	Neer II/Capanna II	NA	NA	NA	Neer II/Capanna II
19*	Neer I/Capanna I	Neer II/Capanna II	Recurrent: Neer IV/Capanna IV see Table 6	—	—	—	—	Recurrent at 2 years; see Table 6
20	Neer I/Capanna I	Neer I/Capanna I	Neer I/Capanna I	Neer II/Capanna II	NA	NA	NA	Neer II/Capanna II
21	Neer I/Capanna I	Neer I/Capanna I	Neer I/Capanna I	Neer I/Capanna I	NA	NA	NA	Neer I/Capanna I
22	Neer I/Capanna I	Neer I/Capanna I	Neer I/Capanna I	Neer I/Capanna II	NA	NA	NA	Neer I/Capanna II
23	Neer I/Capanna I	Neer I/Capanna I	Neer I/Capanna I	Neer II/Capanna I	NA	NA	NA	Neer II/Capanna I
24	Neer I/Capanna I	Neer I/Capanna I	Neer I/Capanna II	Neer II/Capanna II	NA	NA	NA	Neer II/Capanna II
25	Neer I/Capanna I	Neer I/Capanna I	Neer I/Capanna II	Neer I/ Capanna I	Neer I/Capanna II	NA	NA	Neer I/Capanna II
26	Neer I/Capanna I	Neer I/Capanna I	NA	NA	NA	NA	NA	Neer I/Capanna I
27	Neer I/Capanna I	Neer I/Capanna II	Neer I/Capanna II	NA	NA	NA	NA	Neer I/Capanna II
28	Neer I/Capanna I	Neer I/Capanna I	Neer I/Capanna I	Neer I/Capanna I	NA	NA	NA	Neer I/Capanna I
29*	Neer I/Capanna I	Neer I/Capanna I	Neer II/Capanna III	NA	NA	NA	NA	Radiographic recurrence: Neer II/Capanna III
30	Neer I/Capanna I	Neer I/Capanna I	Neer I/Capanna I	Neer I/Capanna I	Neer I/Capanna II	NA	NA	Neer I/Capanna II
31	Neer I/Capanna I	Neer I/Capanna I	NA	NA	NA	NA	NA	Neer I/Capanna I

* Asterisks indicate recurrence after the first injection. Cases 1, 17, and 19 underwent repeat CPC injections, whereas Case 29 was observed; NA, no available image data.

### Recurrence and complications

Recurrences after the first CPC injection occurred in four patients (4/31, 12.9%; 95% CI, 3.6%–29.8%). Three patients underwent repeat percutaneous CPC injections, whereas one patient was managed with observation because of adequate cortical thickness and clinical stability (Table 6). In the exploratory subgroup analysis, recurrence occurred in 1 of 14 patients with preoperative pathological fractures and in 3 of 17 patients without preoperative pathological fracture. Because of the limited sample size and small number of recurrence events, these subgroup results should be interpreted descriptively.

**Table 6:** Management and outcomes of recurrent cases after the first percutaneous calcium phosphate cement injection.

Case	Time to recurrence after first injection	Management after recurrence	Time of repeat injection	1 year after repeat injection	2 years after repeat injection	3 years after repeat injection	Final follow-up after repeat injection	Further recurrence	Additional treatment	Follow-up after repeat injection, months
1	1 year	repeat injection	1 year after first injection	Neer I/Capanna I	Neer I/Capanna I	Neer I/Capanna I	Neer II/Capanna II	No	No	127
17	3 years	repeat injection	3 years after first injection	Neer I/Capanna I	Neer I/Capanna I	Neer I/Capanna I	Neer I/Capanna I	No	No	113
19	2 years	repeat injection	2 years after first injection	Neer I/Capanna I	Neer I/Capanna I	NA	Neer I/Capanna I	No	No	31
29	2 years	Observation	Not performed	NA	NA	NA	NA	No	No	NA

**Figure 3:**
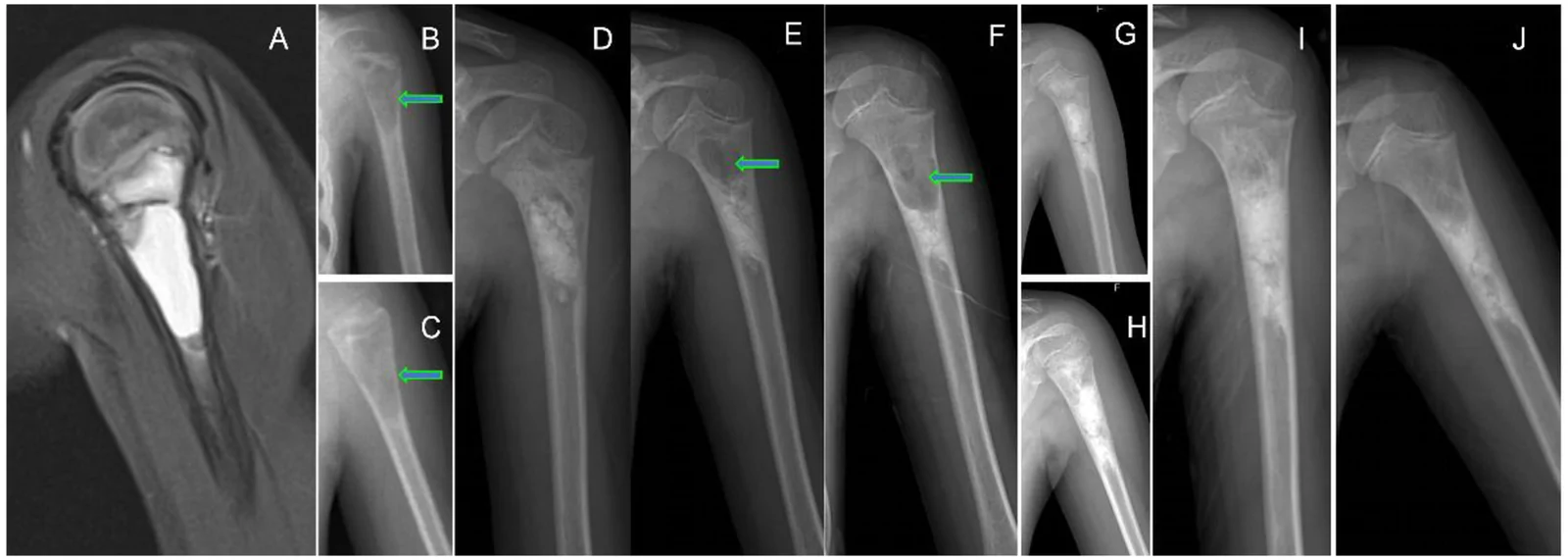
Imaging follow-up of a unicameral bone cyst treated with repeated percutaneous calcium phosphate cement injection. Initial MRI and radiographs showed a proximal humeral cystic lesion **(A–C)**. Follow-up radiographs at 3, 12, and 24 months after the first injection demonstrated progressive cyst filling and remodeling **(D–F)**. After a second injection **(G,H)**, radiographs at 12 and 24 months showed further consolidation without obvious progression **(I,J)**.

Case 1 showed radiographic recurrence 1 year after the first injection and underwent repeat CPC injection. At final follow-up after the second injection, this patient was classified as modified Neer grade II and Capanna grade II, without further recurrence or additional treatment. Case 17 developed recurrence 3 years after the first injection and received repeat CPC injection. During a follow-up period of 113 months after the second injection, this patient maintained modified Neer grade I and Capanna grade I outcomes, with no further recurrence. Case 19 was classified as modified Neer grade II and Capanna grade II 1 year after the first injection, but cyst enlargement was observed 2 years after the first injection, and a second CPC injection was performed. At final follow-up after repeat injection, this patient achieved modified Neer grade I and Capanna grade I, without additional recurrence (Figure 3). Case 29 showed radiographic recurrence 2 years after the first injection. However, the patient had adequate cortical thickness, no progressive symptoms, and no new pathological fracture. Therefore, this case was considered asymptomatic radiographic recurrence rather than clinical failure, and close observation was recommended instead of repeat intervention.

One patient experienced delayed wound healing after surgery, which was resolved after local wound care and supportive treatment. No deep infection, material rejection, symptomatic cement leakage, neurovascular injury, new pathological fracture, or severe adverse event was observed during follow-up.

## Discussion

In this retrospective cohort, we demonstrated that percutaneous CPC injection provided durable clinical improvement and radiographic stabilization in patients with UBCs during mid- to long-term follow-up. In this cohort of 31 patients, most patients achieved substantial pain relief and functional recovery at final follow-up. Radiographically, most lesions showed satisfactory cortical thickening and cavity filling after treatment. Recurrence after the first injection occurred in four patients, three of whom underwent repeat CPC injection and one of whom was managed with observation because of adequate cortical thickness and clinical stability. These findings suggest that percutaneous CPC injections may serve as a minimally invasive treatment option for selected UBC patients, and that repeat injection may be considered in selected recurrent cases.

Mid- to long-term follow-up is important in the evaluation of UBC treatment because early radiographic improvement does not necessarily indicate durable healing. UBCs frequently occur in children and adolescents, and residual cystic lesions may change during skeletal growth. Therefore, the assessment of treatment success should not be limited to early cavity filling or short-term symptom relief. Instead, recurrence, maintenance of cortical thickness, cavity filling, and functional recovery should be evaluated over time. The need for individualized treatment according to cyst size, symptoms, fracture risk, and lesion activity has been emphasized in previous reviews of UBC management (3, 29). In the present study, most patients maintained satisfactory clinical and radiographic outcomes during follow-up, supporting the durability of this minimally invasive approach in appropriately selected patients.

The treatment strategy for UBCs remains controversial (29). Open curettage combined with autologous or allogeneic bone grafting has traditionally been used for symptomatic lesions or lesions with a high risk of fracture, but this approach may require larger surgical exposure and, in some cases, additional fixation (24). Since Scaglietti introduced percutaneous steroid injection for UBCs, injection-based treatment has become an important minimally invasive strategy (25). Other injectable materials, including autologous bone marrow and bone graft substitutes, have also been used for UBC treatment (21, 26–28). However, repeated treatment is often required in injection-based therapy, and radiographic healing may vary among patients. In this context, CPC injections combine cyst decompression, cavity filling, and structural reinforcement, which may be particularly useful for lesions with cortical thinning or a risk of pathological fracture.

The material characteristics of CPC may help explain the radiographic stability observed in this cohort. CPC is an injectable, self-setting calcium phosphate material with good osteoconductivity and biocompatibility. Compared with rapidly resorbable materials such as calcium sulfate, CPC degrades more slowly and may provide more sustained mechanical support during cortical remodeling over longer follow-up. Compared with non-resorbable materials such as PMMA, CPC has better osteoconductivity and does not generate excessive heat during setting (16–19). *In vivo*, CPC resorption is generally a gradual process involving passive dissolution, cell-mediated resorption, vascular ingrowth, osteoconduction, and creeping substitution at the bone–cement interface (30). Because its resorption rate may be influenced by material composition, porosity, implantation site, and the local biological environment, residual CPC may remain radiographically visible for a prolonged period rather than being completely replaced in the short term (31). This feature may be particularly relevant in UBCs with thin cortices, where treatment should not only promote cyst healing but also restore structural stability.

Previous studies using synthetic bone substitutes for benign bone lesions or bone cysts have reported favorable functional outcomes, but repeat intervention, postoperative fracture, wound-related complications, or inflammatory reactions remain concerns (32, 35, 39). A comparison of previous studies using injectable bone substitutes for UBCs or benign bone lesions is summarized in Table 7. In the present study, recurrence after the first CPC injection occurred in 12.9% of patients, and most patients maintained improved cortical thickness ratio and cavity filling at final follow-up, suggesting that CPC may provide sustained structural support during bone remodeling. Previously reported recurrence or repeat-treatment rates were approximately 15%–30% after steroid injection and 20%–25% after bone marrow injection. Because the present study was a single-center, retrospective study without a control group, and because previous studies differed in patient selection, lesion location, follow-up duration, and outcome definitions, a formal statistical comparison could not be performed. Nevertheless, descriptively, the recurrence rate in this cohort appears comparable to or slightly lower than those reported for other injection therapies, suggesting that percutaneous CPC injection may be an acceptable treatment option. Although CPC may have a higher material cost than steroid injection, its minimally invasive nature and ability to combine cyst decompression with structural filling may reduce the need for open curettage, bone grafting, or internal fixation in selected patients.

**Table 7:** Comparison of clinical studies using injectable materials or bone substitutes for UBCs or benign bone lesions.

Study	Year	Lesion type	Treatment approach	Material	No. of patients/follow-up	Main outcomes	Recurrence/repeat treatment	Complications or limitations
D'Amato et al. (15)	2020	UBCs	Percutaneous injection therapy	Bone marrow concentrate with equine-derived demineralized bone matrix versus methylprednisolone acetate	53 patients; minimum follow-up, 24 months	Compared biological injection therapy with steroid injection for UBCs	NR	Did not evaluate CPC or calcium phosphate cement-based structural filling
Huang et al. (32)	2024	Symptomatic bone cysts	Percutaneous microwave ablation and reconstruction	Biomimetic mineralized collagen bone repair material	14 cases; mean follow-up, 28.9 months	Minimally invasive lesion inactivation and reconstruction achieved symptom relief and radiographic improvement	NR	Requires intraoperative temperature control; minor skin burns, tingling, and pathological fracture were reported
Li et al. (33)	2020	UBC of the humerus	ESIN combined with bone grafting	Injectable calcium sulfate or mixed autologous iliac bone and allogeneic bone graft	36 cases; mean follow-up, 3.4 ± 1.3 years	Calcium sulfate and mixed bone graft showed similar healing results	NR	Calcium sulfate alone as isolated percutaneous injection was not evaluated
Zhang et al. (34)	2018	Pediatric bone cysts	Bone graft substitute injection	Triphasic bone graft	41 patients; mean follow-up, 2.8 years	Simple procedure with limited trauma	NR	Pathological fracture and local inflammatory reaction were reported
Fillingham et al. (35)	2012	Benign bone lesions	Bone graft substitute injection	CaSO_4_-CaPO_4_ composite graft material	56 patients; mean follow-up, 42 months	Functional improvement after injection of benign bone lesions	NR	Postoperative fracture and wound complications were reported
Gitelis et al. (36)	2001	Benign bone lesions	Curettage combined with bone graft substitute	Calcium sulfate	23 patients; minimum follow-up, 12 months	Biodegradable and osteoconductive bone void filling	NR	Postoperative fracture was reported
Hirata et al. (37)	2006	Benign bone tumors	Curettage combined with bone graft substitute	Purified beta-tricalcium phosphate	53 patients; mean follow-up, 21 months	Biocompatibility and resorption characteristics for bone defect filling	Local recurrence reported	Focused on defects after curettage rather than injection alone
Kelly and Wilkins (38)	2004	Benign bone lesions	Curettage combined with bone graft substitute	Injectable calcium sulfate-based bone graft substitute	15 patients; mean follow-up, 6 months	Used for filling defects after treatment of benign bone lesions	NR	Infection was reported
Doring et al. (39)	2022	Benign bone lesions	Curettage with bone substitute or percutaneous treatment	Injectable biphasic ceramic bone substitute	11 percutaneous injection cases; median follow-up, 36.5 months	Radiographic bone mineralization and osteoconductive potential	NR	Wound exudation and residual soft-tissue material were reported
Fillingham et al. (40)	2016	UBCs	Percutaneous bioceramic injection	75% CaSO_4_ and 25% DCPD/*β*-TCP granules	12 patients; mean follow-up, 41 months	Bioceramic substitute with triphasic resorption profile	Repeating percutaneous injection was required in some cases	Repeat treatment remained necessary in selected patients
Present study	-	UBCs	Percutaneous CPC injection with aspiration and saline lavage	Calcium phosphate cement	31 patients; mean follow-up, 61.1 months	Pain relief, functional recovery, cortical remodeling, and cavity filling during follow-up	Recurrence after first injection occurred in 4/31 patients; 3 underwent repeat CPC injection and 1 was observed	One delayed wound healing; no deep infection, material rejection, symptomatic cement leakage, neurovascular injury, or new pathological fracture

NR is not reported or not clearly specified.

Recurrence after CPC injection did not necessarily lead to open surgery in this cohort. Repeat percutaneous CPC injections appeared to provide stable outcomes in selected recurrent cases, whereas close observation was considered reasonable for one clinically stable lesion with adequate cortical thickness. These findings suggest that radiographic recurrence may not always be equivalent to clinical failure. The management of recurrence should be individualized according to radiographic progression, clinical symptoms, cortical support, activity limitation, and fracture risk. For progressive or symptomatic recurrence, especially when cyst enlargement, cortical thinning, or increased fracture risk is present, repeat intervention may be considered; for asymptomatic and structurally stable lesions, close observation may be an appropriate option.

Patients with preoperative pathological fracture represent an important subgroup in UBC treatment. In this cohort, patients with pathological fracture were initially managed with immobilization or fixation when necessary, before CPC injection. Most of these patients achieved satisfactory pain relief and functional recovery after treatment. Pathological fracture may alter the biological and mechanical environment of the cyst, but its influence on subsequent healing remains uncertain. In our cohort, the presence of a previous pathological fracture did not preclude favorable clinical outcomes after CPC injection. However, because the number of recurrent cases was small, the relationship between pathological fracture and recurrence should be interpreted cautiously.

Several technical points may be important for achieving favorable outcomes. First, the diagnosis should be established by combining clinical presentation, radiographs, CT, MRI, and pathological confirmation when necessary. Second, accurate puncture under C-arm fluoroscopic guidance is essential to ensure access to the cyst cavity while minimizing unnecessary cortical damage. Third, the use of two puncture channels allows aspiration and saline lavage, which may help remove cyst fluid and reduce intracystic pressure. Fourth, CPC should be injected gradually under fluoroscopic monitoring to achieve adequate cavity filling while avoiding leakage into the surrounding soft tissue or adjacent joint space. These steps are particularly important for lesions near the physis, in weight-bearing bones, or in patients with marked cortical thinning.

This study has several limitations. First, it was a retrospective single-center study with a relatively small sample size and no control group, which may limit the generalizability of the findings and precluded direct comparisons with curettage, steroid injection, bone marrow injection, calcium sulfate-based substitutes, or conservative treatment. Second, the follow-up intervals were not completely uniform, and some patients underwent radiographic follow-up at local hospitals, which may have introduced variability in image quality and radiographic assessment. Third, because only a limited number of recurrence events occurred, recurrence-related factors and subgroup comparisons could only be interpreted descriptively. The *post-hoc* sensitivity analysis also indicated that the present study had limited statistical power and that smaller but clinically meaningful differences may not have been detected.

In addition, although radiographic measurements were performed by two experienced radiologists and further reviewed by two orthopedic oncology specialists, formal intra-observer reliability analysis based on repeated blinded measurements and formal inter-observer reliability statistics were not available because observer-specific measurement datasets were not separately recorded in this retrospective study. In addition, cyst volume was calculated using the ellipsoid formula based on plain radiographs and should therefore be regarded as an estimated radiographic volume rather than an exact three-dimensional volume. CT-based volumetry would provide more accurate assessment of cyst size in future studies. Detailed economic data, including hospitalization cost, operative time, material cost, rehabilitation cost, and cost of repeat treatment, were also not systematically collected; therefore, a formal cost-effectiveness analysis could not be performed. Further multicenter prospective comparative studies with larger cohorts, standardized follow-up protocols, formal observer reliability analysis, and health economic evaluation are needed to clarify the indications, relative advantages, long-term durability, and cost-benefit profile of percutaneous CPC injection for UBCs.

## Conclusion

Percutaneous CPC injection was associated with pain relief, functional improvement, and radiographic stabilization in patients with UBCs. Repeat CPC injection appeared to provide stable outcomes in recurrent cases, whereas observation was considered reasonable for one clinically stable lesion with adequate cortical thickness. The observed cortical remodeling and cavity filling suggest that CPC injection may represent a potential minimally invasive strategy for reconstruction of UBC-related bone defects. However, because this was a retrospective single-arm study without a control group, these findings should be considered exploratory and require validation in future prospective comparative studies.

## Data Availability

The original contributions presented in the study are included in the article/Supplementary Material, further inquiries can be directed to the corresponding author.
